# Clinical Analysis of Fetal Lung Development Index and Pregnancy Outcome in Pregnant Women with Gestational Diabetes Mellitus with Satisfactory Blood Glucose Control

**DOI:** 10.1155/2022/5777804

**Published:** 2022-09-19

**Authors:** Ting Han, Xiao-dan Jin, Ju-fang Yang, Yan Tang

**Affiliations:** ^1^Maternal Health Care Department, Hunan Maternal and Child Health Hospital, Changsha, Hunan, China; ^2^Ultrasound Department, Hunan Maternal and Child Health Hospital, Changsha, Hunan, China; ^3^Medical Section, Hunan Maternal and Child Health Hospital, Changsha, Hunan, China; ^4^Health Management Center, Hunan Maternal and Child Health Hospital, Changsha, Hunan, China

## Abstract

**Objective:**

To explore the regularity of fetal lung development of pregnant women with gestational diabetes mellitus (GDM) with satisfactory blood glucose control and the clinical analysis with pregnancy outcome.

**Methods:**

120 GDM pregnant women with satisfactory blood glucose control (GDM group) and 200 normal pregnant women undergoing prenatal examination (Control group) from 31 to 38 ^+ 6^ weeks of gestation were included. The two groups of pregnant women were divided into 8 time periods according to the gestational age, respectively. The parameters of Doppler flow velocity curve of fetal main pulmonary artery, diameter lines of fetal lung development, mode of delivery, neonatal weight, neonatal asphyxia, neonatal respiratory distress syndrome (NRDS), and neonatal pneumonia were, respectively, compared and analyzed between the two groups.

**Results:**

Acceleration time (AT) and AT/ejection time (AT/ET) were positively correlated with gestational age, and AT/ET showed stronger correlation than AT, while no significant difference in AT and AT/ET between the two groups (*P* > 0.05). There was a positive correlation between the diameter of fetal lung development and gestational age, but there was no significant difference between the two groups (*P* > 0.05). In addition, there was no significant difference between the two groups in fetal delivery mode, neonatal weight, neonatal asphyxia, NRDS, and neonatal pneumonia (*P* > 0.05).

**Conclusion:**

AT/ET may be a potential index to evaluate fetal lung maturity. There was no difference in fetal lung development and neonatal birth outcome between pregnant women with satisfactory GDM blood glucose control and the normal pregnant women. The pregnancy of GDM pregnant women lasts until the end of 37∼38 weeks, and the neonatal incidence rate is decreased. The key is to manipulate the blood glucose in the normal range.

## 1. Introduction

Gestational diabetes mellitus (GDM) is a common pregnancy complication affecting the health of mothers and infants, which is a crucial inducing factor for neonatal respiratory distress syndrome (NRDS) as well as a variety of adverse pregnancy outcomes, affecting the growth and development of the fetus and leading to adverse pregnancy outcomes such as abnormal fetus, premature birth, macrosomia, intrauterine distress, fetal asphyxia, and neonatal hypoglycemia [1, 2]. Studies have concluded a spiraling rate of about 5% in the incidence rate of pregnancy complicated with diabetes in the world [3], and as high as 15% in China. Therefore, analyzing its impact on fetal growth and development is essential and premier for avoiding adverse pregnancy outcomes and enhancing the birth quality of newborns.

Robert et al. found that the risk of NRDS in pregnant women with GDM is six times higher than that in normal pregnant women, whose greatest impact of GDM on the fetus is the delay of fetal lung maturity [4]. Accurate prenatal evaluation of fetal lung maturity can help obstetricians accurately grasp the delivery opportunity of pregnant women and reduce the incidence of NRDS [5]. Especially in the face of obstetric emergencies such as premature delivery, premature rupture of membranes, and placenta previa, the determination of fetal lung maturity can reduce the perinatal incidence rate and mortality, and plays a vital role in improving the quality of obstetrics [6]. At present, the clinical evaluation of fetal lung maturity is mainly based on amniotic fluid aspiration and analysis of lecithin/sphingomyelin (L/S) ratio and lamellar body count (LBC) [7, 8]. Because amniocentesis is an invasive operation, which has operational risks and accidents, and is difficult for pregnant women to accept, the application of prenatal assessment of fetal lung maturity in clinical work is very limited [9, 10]. Therefore, it is urgent to develop a noninvasive, safe, and efficient method for prenatal assessment of fetal lung maturity to meet the actual clinical needs.

Understanding the growth law of fetal lung and fetal pulmonary circulation is the basis for evaluating fetal lung maturity [11]. Although the tissue structure of fetal lung did not develop until 36 weeks, the outline of fetal lung could be clearly displayed in the second trimester of pregnancy. The growth law of fetal lung could be reflected by measuring the diameter lines of fetal lung through imaging examination [12]. With the development of color Doppler flow imaging technology, it is gradually used to evaluate fetal pulmonary circulation (mainly pulmonary artery flow) [13, 14]. In order to acquire a more objective basis, this subject intends to compare the differences of fetal main pulmonary artery Doppler flow curve parameters, fetal lung development diameter lines, and neonatal birth outcomes between GDM pregnant women with satisfactory blood glucose control and normal pregnant women, exploring the law of fetal lung development in the two groups and the correlation between fetal main pulmonary artery Doppler flow curve parameters and fetal lung maturity, and determining the most significant parameters, so as to reflect the maturity of fetal lung development. It provides a noninvasive method for clinicians to evaluate fetal lung maturity, and also serves as a reference basis for clinical management of GDM pregnant women and grasping the time point of delivery.

## 2. Data and Methods

### 2.1. Research Object and Grouping

160 singleton pregnant women conducting regular prenatal visits and inpatient deliveries in our hospital from October 2020 to December 2021 and diagnosed with GDM before 28 weeks of gestation and satisfactory blood glucose control were selected as the GDM group. 213 normal singleton pregnant women undergoing prenatal examination and delivered in hospital in the same period were selected as the control group. All pregnant women voluntarily accepted the examination on the premise of informed consent. This study was approved by the ethics committee of the Hunan Maternal and Child Health Hospital (2020-S080).

Inclusion criteria (GDM group): ① pregnant women are healthy with accurate and regular menstruation and have no bad habits and other diseases; ② Early ultrasound examination of the gestational week of the fetus confirmed to the actual gestational week, and structural malformations were excluded by ultrasound system screening during pregnancy; ③ GDM pregnant women had satisfactory blood glucose control from 31 weeks to termination of pregnancy. ④ Single live fetus.

Exclusion criteria (GDM group): ① abnormal blood glucose found in the process of test; ② Failure to satisfactorily display the Doppler flow spectrum of fetal right lung and main pulmonary artery due to maternal obesity or other reasons; ③ The presence of large artery malformations or other malformations which may affect fetal respiratory function.

Inclusion criteria (Control group): ① pregnant women are healthy with accurate and regular menstruation and have no bad habits and other diseases; ② Early ultrasound examination of the gestational week of the fetus confirmed to the actual gestational week, and structural malformations were excluded by ultrasound system screening during pregnancy; ③ Single live fetus.

Exclusion criteria (Control group): ① fetal malformations possibly affecting respiratory function or large artery malformations and other serious cardiac malformations; ② The Doppler flow spectrum of fetal right lung and main pulmonary artery fail to be displayed satisfactorily due to maternal obesity or other reasons.

**Figure 1:**
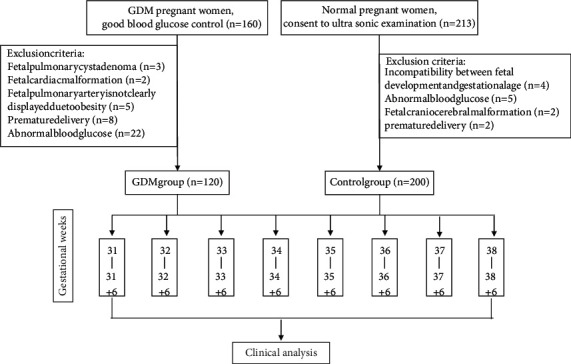
Flowchart of participant inclusion.

According to the screening criteria, 120 pregnant women were included in the GDM group and 200 were in the control group. The age of pregnant women in the GDM group was 26∼37 years, and the blood glucose was 4.3∼5.2 mmol/L. The age of pregnant women in the control group was 25∼36 years, and the blood glucose was 3.7∼4.9 mmol/L. The two groups of pregnant women were divided into 8 time periods according to the gestational age from 31 to 38 ^+ 6^ weeks, respectively. 15 persons in the study group and 25 persons in the control group were selected at each time period.  Figure 1 shows the inclusion process of the participants.

### 2.2. Diagnostic Criteria for GDM

The diagnostic criteria for GDM refer to the health industry standard of the people's Republic of China WS 331-2011, diagnosis of gestational diabetes [15]. Pregnant women routinely underwent 75 g glucose tolerance test (OGTT) at point in 24∼28 weeks of pregnancy. Patients with abnormal blood glucose at any point are supposed to be diagnosed as GDM. The whole blood fasting or premeal blood glucose ≤5.3 mmol/L, and whole blood glucose ≤6.7 mmol/L 2 hours after meal were defined as the criteria for satisfactory blood glucose control of pregnant women with GDM.

### 2.3. Instruments and Methods

Firstly, the fetal systems were screened to eliminate malformations, and the fetal size was determined to be in line with the normal gestational week. Anatomically, the left lung is more likely to be affected by the heart and prone to deviation in the process of measurement, so the right lung is selected to observe the indicators of fetal lung development.

**Figure 2:**
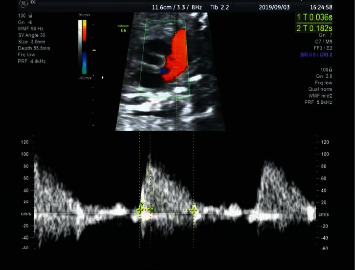
Measurement methods of acceleration time (AT) and ejection time (ET).

Measurement of Doppler parameters: the short axis section of fetal heart bottom was taken, the bimodal fetal pulmonary artery flow velocity curve was obtained(Figure 2), acceleration time (AT) and ejection time (ET) were measured by manual spectrum profilometry, with the AT/ET ratio calculated. They were measured at least three times and the average value was taken.

**Figure 3:**
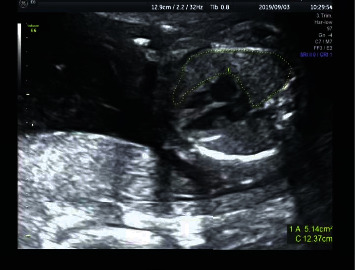
Fetal lung development index: right lung area.

**Figure 4:**
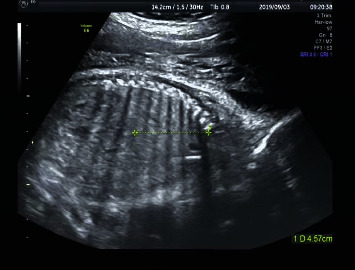
Upper and lower diameter of the right lung.

Measurement of fetal lung development index: the standard four chamber cardiac section was taken, and the area of right lung, chest area (excluding spine), anterior posterior diameter of right lung and left and right diameter of right lung at the end of diastole (Figure 3) were manually outlined; the sagittal section of the right lung was taken and the upper and lower diameters of the right lung were measured (Figure 4).

### 2.4. Maternal and Infant Follow-Up Records

1. Record the basic information: age, gestational week of delivery, mode of delivery and neonatal weight
2. Record the neonatal condition: neonatal hypoglycemia, neonatal pneumonia, NRDS, neonatal asphyxia, neonatal death, and other indicators

### 2.5. Statistical Analysis

All the statistical analyses were performed by using SPSS22.0 software. The statistical description of measurement data was mean ± standard deviation (SD) and Student's *t*-test was used for the comparison *χ*^2^ test was utilized to compare the rate counting data, *P* < 0.05 means the difference is statistically significant.

## 3. Result

### 3.1. The Basic Conditions of Pregnant Women

There was no significant difference in the age of pregnant women, gestational weeks of delivery, mode of delivery, and neonatal birth weight between the two groups (*P* > 0.05, Table 1).

### 3.2. Changes of Doppler Parameters with Gestational Age in the GDM Group

Doppler parameters in the GDM group are shown in Table 2, where AT and AT/ET were associated with an increase with gestational week. The analysis of variance of Doppler parameters at different gestational weeks showed a significant difference between AT and AT/ET (*F* = 43.175 and 108.171, *P* < 0.05), while parameter ET has no obvious correlation (*F* = 8.625, *P* > 0.05).

### 3.3. Changes of Doppler Parameters with Gestational Age in the Control Group

Doppler parameters in the control group are shown in Table 3. The parameters AT and AT/ET exhibit an increase with gestational weeks, with no correlation between ET and gestational age. Analysis of variance of Doppler parameters at different gestational weeks showed a significant difference in AT and AT/ET (*F* = 68.137 and 128.064, *P* < 0.05), but ET has no obvious correlation (*F* = 4.825, *P* > 0.05).

### 3.4. Comparison of the Doppler Parameters of Fetal Main Pulmonary Artery between the Two Groups

Table 4 depicts the comparison of fetal primary pulmonary artery Doppler parameters between the two groups. The results showed that the Doppler parameters AT, ET, and AT/ET have no significant difference between the two groups in any gestational weeks (*P* > 0.05).

### 3.5. Changes of Fetal Lung Development Indexes with Gestational Weeks in the GDM Group

The measured values of fetal lung development indexes in the GDM group are listed in Table 5. All the measured values of fetal lung development indexes were positively correlated with gestational weeks, and display significant differences among fetuses at different gestational weeks (*P* < 0.05).

### 3.6. Changes of Fetal Lung Development Indexes with Gestational Weeks in the Control Group

The measured values of fetal lung development indexes in the control group are shown in Table 6. All the measured values of fetal lung development indexes were also positively correlated with gestational weeks, and display significant differences among fetuses at different gestational weeks (*P* < 0.05).

### 3.7. Comparison of Neonatal Outcomes between the Two Groups

The incidence of NRDS, neonatal pneumonia, neonatal hypoglycemia, and neonatal asphyxia in both groups were poor, without statistically significant differences (*P* > 0.05), appearing no neonatal death in both groups (Table 7).

## 4. Discussion

In the present study, the results showed a positive correlation of AT and AT/ET with the gestational age in both GDM with satisfactory blood glucose control and normal pregnant women where all increased with gestational age, reflecting an increase in pulmonary vascular compliance and a decrease in the mean pulmonary arterial pressure. Theoretically, with the increase of gestational age, the number of pulmonary artery vascular beds increases and Pap gradually decreases, indicating that the results of this study are consistent with the development of fetal lung in theory, AT and AT/ET can serve as reliable Doppler velocity indicators for evaluating fetal pulmonary circulation, and AT/ET shows stronger correlation than at here, which is consistent with the published research results [16–18]. AT/ET may be a potential and ideal index for noninvasive prenatal evaluation of FLM.

There exists a long debate on the influence of diabetes pregnancy on fetal lung maturation, mainly focusing on: will diabetes cause the delay of fetal lung maturation? Is blood glucose control in pregnant women with diabetes related to fetal lung maturation [19, 20]? This study shows no difference in the incidence of NRDS and neonatal asphyxia between diabetes pregnant women with satisfactory blood glucose control and normal pregnant women, indicating that a close relationship between the blood glucose control level of pregnant diabetes pregnant women and the maternal and infant outcomes, and diabetes pregnant women with satisfactory blood glucose control will not lead to the delay of fetal lung maturation. Therefore, early screening and active treatment to control the disease and manipulate the blood glucose level within the normal range have great clinical value in preventing maternal complications and improving perinatal outcomes.

For a long time, the clinical guidelines for the timing of termination of pregnancy for pregnant women with GDM refer to the traditional diagnostic criteria of GDM: for GDM patients treated with insulin, it is recommended to control blood glucose until 38∼39 weeks and then terminate the pregnancy; for GDM patients free from insulin therapy, it is recommended to observe until 40 weeks then terminate pregnancy. However, whether the new diagnostic criteria of GDM should be utilized still remains controversial. This study shows no significant difference in the gestational weeks of delivery, neonatal incidence rate, perinatal mortality, and the incidence of macrosomia between the GDM pregnant women satisfied with blood glucose control depending on reasonable diet and exercise and those of normal pregnant women, indicating a possibility that the gestation of GDM pregnant women lasts until the 37th to 38th weeks to reduce the neonatal morbidity, with the crux of controlling blood glucose within the normal range.

In conclusion, pregnant women with GDM who have satisfactory glycemic control do not differ from normal pregnant women in fetal lung development and neonatal birth outcomes. Diabetes screening and examination should be accepted as soon as possible during pregnancy, with the key to ensuring health education guidance and monitoring during GDM pregnancy. Manipulating blood glucose within the standard range can reduce the risk of adverse perinatal outcomes.

## Figures and Tables

**Table 1:** Comparison of basic conditions between the two groups.

Group	GDM group (*n* = 120)	Control group (*n* = 200)	*T*	*P* value
Age (year)	31.3 ± 5.1	30.2 ± 4.9	−0.121	0.795
Gestational week of delivery	37.8 ± 0.6	37.2 ± 0.5	1.124	0.192
Mode of delivery (cesarean section rate)	37 (31.16)	57 (28.69)	0.153	0.234
Neonatal weight (g)	3322.23 ± 143.2	3298.54 ± 171.1	0.171	0.215

**Table 2:** Comparison of Doppler flow velocity parameters in pregnant women with gestational diabetes mellitus.

Group	Gestational weeks	N	AT (ms)	ET (ms)	AT/ET
1	31–31^+6^	15	38.23 ± 4.12	177.79 ± 14.23	0.208 ± 0.016
2	32–32^+6^	15	39.13 ± 4.25	180.34 ± 11.38	0.218 ± 0.021
3	33–33^+6^	15	40.34 ± 3.78	179.82 ± 12.68	0.229 ± 0.012
4	34–34^+6^	15	41.52 ± 3.34	179.56 ± 11.32	0.231 ± 0.011
5	35–35^+6^	15	42.68 ± 3.49	178.81 ± 15.13	0.239 ± 0.023
6	36–36^+6^	15	44.72 ± 2.33	180.33 ± 11.61	0.241 ± 0.033
7	37–37^+6^	15	46.23 ± 3.35	180.26 ± 12.22	0.251 ± 0.019
8	38–38^+6^	15	46.82 ± 8.12	181.05 ± 10.36	0.257 ± 0.026
F			43.175	8.625	108.171
*P* value			0.023	0.735	0.009

**Table 3:** Comparison of Doppler flow velocity parameters in the control group of pregnant women with different gestational weeks.

Group	Gestational weeks	N	AT (ms)	ET (ms)	AT/ET
1	31–31^+6^	25	38.83 ± 4.02	178.72 ± 10.23	0.216 ± 0.019
2	32–32^+6^	25	39.66 ± 4.12	180.92 ± 10.12	0.225 ± 0.029
3	33–33^+6^	25	41.32 ± 2.23	180.22 ± 10.68	0.231 ± 0.016
4	34–34^+6^	25	41.62 ± 2.34	180.56 ± 9.32	0.234 ± 0.014
5	35–35^+6^	25	43.26 ± 3.99	179.81 ± 10.13	0.241 ± 0.023
6	36–36^+6^	25	45.22 ± 3.18	181.13 ± 11.61	0.245 ± 0.198
7	37–37^+6^	25	46.03 ± 3.35	180.56 ± 11.36	0.250 ± 0.023
8	38–38^+6^	25	46.54 ± 4.12	181.45 ± 9.34	0.255 ± 0.022
F			68.137	4.825	128.064
*P* value			0.011	0.827	0.006

**Table 4:** Comparison of Doppler parameters of fetal main pulmonary artery between the two groups.

Gestational weeks		AT (ms)	ET (ms)	AT/ET

31–31^+6^	T	8.023	5.238	13.242
	P	0.254	0.423	0.132

32–32^+6^	T	6.547	3.942	12.132
	P	0.486	0.521	0.243

33–33^+6^	T	5.124	3.432	10.265
	P	0.532	0.611	0.335

34–34^+6^	T	3.243	2.657	9.465
	P	0.597	0.638	0.413

35–35^+6^	T	1.738	2.123	8.967
	P	0.797	0.715	0.638

36–36^+6^	T	1.533	1.874	8.226
	P	0.802	0.756	0.654

37–37^+6^	T	1.213	1.324	6.325
	P	0.847	0.833	0.713

38–38^+6^	T	0.823	0.125	1.634
	P	0.839	0.908	0.806

**Table 5:** Comparison of lung development indexes of fetuses in pregnant women with gestational diabetes mellitus.

Group	Gestational weeks	N	RLa	Ta	Upper and lower diameter	Left and right diameter	Anterior and posteriordiameter
1	31–31^+6^	15	10.72 ± 1.09	29.69 ± 3.01	4.44 ± 0.47	2.53 ± 0.27	6.26 ± 0.40
2	32–32^+6^	15	10.99 ± 1.56	30.80 ± 2.70	4.59 ± 0.46	2.58 ± 0.25	6.40 ± 0.379
3	33–33^+6^	15	11.14 ± 2.07	31.61 ± 2.55	4.60 ± 0.43	2.73 ± 0.24	6.52 ± 0.38
4	34–34^+6^	15	11.96 ± 1.15	32.17 ± 1.81	4.68 ± 0.41	2.95 ± 0.24	6.71 ± 0.36
5	35–35^+6^	15	12.40 ± 1.36	35.13 ± 2.81	4.71 ± 0.42	3.04 ± 0.23	6.93 ± 0.36
6	36–36^+6^	15	13.36 ± 2.24	37.10 ± 2.35	4.88 ± 0.49	3.10 ± 0.23	7.09 ± 0.36
7	37–37^+6^	15	14.02 ± 1.75	38.65 ± 1.56	4.98 ± 0.45	3.20 ± 0.22	7.24 ± 0.31
8	38–38^+6^	15	14.30 ± 2.03	39.26 ± 1.15	5.06 ± 0.44	3.39 ± 0.17	7.31 ± 0.34
F			36.852	164.119	35.499	111.347	182.359
*P* Value			0.003	0.001	0.003	0.002	0.001

**Table 6:** Measurement results of lung development indexes in the control group of pregnant women.

Group	Gestational weeks	N	RLa	Ta	Upper and lower diameter	Left and right diameter	Anterior and posteriordiameter
1	31–31 + 6	25	10.28 ± 1.02	28.91 ± 2.46	4.41 ± 0.57	2.48 ± 0.27	6.19 ± 0.33
2	32–32 + 6	25	10.69 ± 1.34	30.22 ± 2.15	4.46 ± 0.43	2.52 ± 0.23	6.33 ± 0.26
3	33–33 + 6	25	10.94 ± 1.62	30.53 ± 2.57	4.48 ± 0.42	2.65 ± 0.19	6.49 ± 0.29
4	34–34 + 6	25	11.23 ± 1.54	31.56 ± 2.38	4.59 ± 0.45	2.74 ± 0.22	6.59 ± 0.26
5	35–35 + 6	25	12.33 ± 2.12	35.56 ± 3.68	4.68 ± 0.16	2.98 ± 0.18	6.88 ± 0.21
6	36–36 + 6	25	13.12 ± 2.11	37.08 ± 2.14	4.85 ± 0.35	3.02 ± 0.17	7.01 ± 0.24
7	37–37 + 6	25	13.88 ± 1.51	38.33 ± 1.85	4.93 ± 0.25	3.18 ± 0.16	7.22 ± 0.33
8	38–38 + 6	25	14.21 ± 2.24	38.82 ± 1.23	5.01 ± 0.21	3.24 ± 0.13	7.31 ± 0.12
F			22.831	178.031	44.972	105.741	163.185
*P* Value			0.002	≤0.001	0.002	0.001	0.001

**Table 7:** Comparison of neonatal outcomes.

Group	GDM group (*n* = 120)	Control (*n* = 200)	*t*	*P* Value
Neonatal respiratory distress syndrome (NRDS)	3 (2.500)	3 (1.500)	0.962	0.910
Neonatal pneumonia	8 (6.670)	11 (5.550)	0.921	0.891
Neonatal hypoglycemia	7 (5.830)	9 (4.500)	0.954	0.881
Neonatal asphyxia	6 (5.00)	11 (5.550)	0.972	0.923
Neonatal death	0 (0.000)	0 (0.000)	—	—

## Data Availability

The data used to support the findings of this study are available from the corresponding author upon request.

## References

[1] Landon M. B., Mele L., Spong C. Y. (2011). The relationship between maternal glycemia and perinatal outcome. *Obstetrics & Gynecology*.

[2] Szmuilowicz E. D., Josefson J. L., Metzger B. E. (2019). Gestational diabetes mellitus. *Endocrinology and Metabolism Clinics of North America*.

[3] Editorial L. (2008). The global challenge of diabetes. *The Lancet*.

[4] Robert M. F., Neff R. K., Hubbell J. P., Taeusch H. W., Avery M. E. (1976). Association between maternal diabetes and the respiratory-distress syndrome in the newborn. *New England Journal of Medicine*.

[5] Marinov B., Jekova N., Andreeva A., Hitrova S. (2011). [Antenatal ambroxol administration for prevention of respiratopry distress syndrome in preterm infants: preliminary report]. *Akusherstvo I Ginekologiya*.

[6] Piper J. M. (2002). Lung maturation in diabetes in pregnancy: if and when to test. *Seminars in Perinatology*.

[7] Wijnberger L. D. E., de Kleine M., Voorbij H. A. M., ArabinBruinseVisserBossuytMol B. W. J. (2010). Prediction of fetal lung immaturity using gestational age, patient characteristics and fetal lung maturity tests: a probabilistic approach. *Archives of Gynecology and Obstetrics*.

[8] Besnard A. E., Wirjosoekarto S. A., Broeze K. A., Opmeer B. C., Mol B. W. J. (2013). Lecithin/sphingomyelin ratio and lamellar body count for fetal lung maturity: a meta-analysis. *European Journal of Obstetrics & Gynecology and Reproductive Biology*.

[9] Grenache D. G., Gronowski A. M. (2006). Fetal lung maturity. *Clinical Biochemistry*.

[10] López Sánchez F., Delgado Sánchez E., Duyos Mateo I., González ÁlvarezAntolín AlvaradoBartha J. (2019). Evaluation of fetal lung maturity by quantitative analysis (quantusFLM) in women with gestational diabetes mellitus. *Fetal Diagnosis and Therapy*.

[11] Kitterman J. A. (1988). Physiological factors in fetal lung growth. *Canadian Journal of Physiology and Pharmacology*.

[12] Kasprian G., Balassy C., Brugger P. C., Prayer D. (2006). MRI of normal and pathological fetal lung development. *European Journal of Radiology*.

[13] Gagnon R., Van den Hof M. (2003). The use of fetal Doppler in obstetrics. *Journal of Obstetrics and Gynaecology Canada*.

[14] Cynober E., Cabro D., Haddad B., GabrielMorettiGamghuiJeny R. (1997). Fetal pulmonary artery Doppler waveform: a preliminary report. *Fetal Diagnosis and Therapy*.

[15] Gabbe S. G., Persson B. (2010). International association of diabetes and pregnancy study groups recommendations on the diagnosis and classification of hyperglycemia in pregnancy. *Diabetes Care*.

[16] Thibault H. B., Kurtz B., Raher M. J., ShaikWaxmanDerumeauxHalpernBlochScherrer-Crosbie M. (2010). Noninvasive assessment of murine pulmonary arterial pressure. *Circulation: Cardiovascular Imaging*.

[17] Yared K., Noseworthy P., Weyman A. E., McCabePicardBaggish A. L. (2011). Pulmonary artery acceleration time provides an accurate estimate of systolic pulmonary arterial pressure during transthoracic echocardiography. *Journal of the American Society of Echocardiography*.

[18] Chaoui R., Taddei F., Rizzo G., BastLenzBollmann R. (1998). Doppler echocardiography of the main stems of the pulmonary arteries in the normal human fetus. *Ultrasound in Obstetrics and Gynecology*.

[19] Langer O. (2002). The controversy surrounding fetal lung maturity in diabetes in pregnancy: a re-evaluation. *Journal of Maternal-Fetal and Neonatal Medicine*.

[20] Mimouni F., Miodovnik M., Whitsett J. A., HolroydeSiddiqiTsang R. C. (1987). Respiratory distress syndrome in infants of diabetic mothers in the 1980s: no direct adverse effect of maternal diabetes with modern management. *Obstetrics & Gynecology*.

